# Gastrointestinal nematode larvae in the grazing land of cattle in Guwahati, Assam

**DOI:** 10.14202/vetworld.2016.1343-1347

**Published:** 2016-12-03

**Authors:** Meena Das, D. K. Deka, S. Islam, P. C. Sarmah, K. Bhattacharjee

**Affiliations:** 1 Division of Animal Health, ICAR Research Complex for NEH Region, Umiam, Meghalaya, India; 2 Department of Parasitology, College of Veterinary Science, Guwahati, Assam, India

**Keywords:** Assam, cattle, Guwahati, nematode larvae (L_3_), pastures

## Abstract

**Aim:**

To know the prevalence of gastrointestinal nematode larvae (L_3_) in the grazing land of cattle in Guwahati, Kamrup district, Assam.

**Materials and Methods:**

Pastures were collected and examined for the presence of nematode larvae (L_3_) from six localities of Guwahati at monthly interval from August 2012 to July 2013. The counted larvae were then expressed as per kg dry matter of herbage (L_3_/kg DM).

**Results:**

Examination of pastures revealed presence of nematode larvae (L_3_) in pastures throughout the year which varied from 4.5 L_3_/kg DM in January to a maximum of 106.33 L_3_/kg DM in August. The L_3_ of *Haemonchus contortus*, *Trichostrongylus* spp., *Oesophagostomum* spp., *Cooperia* spp., and *Mecistocirrus* spp. were recovered from pastures. The average pasture larval burden (PLB) was 34.75±3.48 L_3_/kg DM. Season-wise PLB revealed the presence of 23.89±3.01, 67.54±5.41, 26.67±1.92, and 7.28±0.89 L_3_/kg DM during pre-monsoon, monsoon, post-monsoon, and winter seasons, respectively. Monsoon season has significant (p<0.05) effect on PLB. However, analysis of variance of different locations with respect to season revealed that there was no significant difference but season-wise it was highly significant (p<0.01). Pearson correlation of environmental variables (temperature, relative humidity, and rainfall) with PLB revealed correlation was statistically significant with rainfall (p<0.05).

**Conclusion:**

This study reveals the presence of five nematode larvae (L_3_) in the pastures of Guwahati, Assam throughout the year, statistically significant during monsoon season.

## Introduction

Livestock plays an important role in Indian economy and is an important subsector of Indian Agriculture. Among the livestock population, cattle (190.90 million) plays a major role in India’s economy, accounting 37.28% of total livestock population [1]. However, as per estimation record of State Animal Husbandry and Veterinary Department, Assam has 8,938,760 cattle population [2]. Gastrointestinal nematode infections in cattle are a worldwide problem for both small and large-scale farmers and are a great threat to livestock industry [3]. The economic losses are mainly due to subclinical effects which go unnoticed to the owners. Subclinical infections are responsible for high morbidity and mortality in young animals and enormous production losses in adults. The economic losses occur in terms of lowered fertility, reduced work capacity, reduction in food efficiency and lower weight gain, lower milk production, increased treatment cost, and mortality in heavily parasitized animals [4]. Susceptible animal gets infection by ingestion of infective nematode larvae (L_3_) along with contaminated pasture.

Assessment of infective larvae in pasture will give an idea of infection in animals grazed in a particular pasture land. Pasture herbage counts of infective larvae are used increasingly in the diagnosis and prognosis of parasitic disease in farm animals [5]. Management of pasture is an important component of nematode parasite control programs because it has several benefits in relation to productivity including weight gain, improved feed conversion, increased milk production, better reproductive performance, greater carcass quality, improved immunological status, and reduced morbidity and mortality [6,7].

Since contaminated pasture is the source of infection, so it is important to know the prevalence of infective larvae in the pasture for adopting suitable grazing practices. Therefore, this study was designed to know the prevalence of nematode larvae in the grazing land of cattle in Guwahati, Assam.

## Materials and Methods

### Ethical approval

The experiments comply with the guidelines laid down by the Institutional Ethical Committee and in accordance with the country law.

### Study area

This study was conducted in Guwahati, the capital city of the state of Assam that lies within the latitude of 26°11’0″N and longitude 91°44’0″E. The city is situated on an undulating plain with varying altitudes of 49.5-55.5 m above mean sea level. The southern and eastern sides of the city are surrounded by hillocks.

### Study period

The study was conducted for one calendar year from August 2012 to July 2013 and divided into four seasons, *viz*., pre-monsoon (March, April, and May), monsoon (June, July, August, and September), post-monsoon (October, November), and Winter (December, January, February).

### Study method

Pasture samples (approximately 250 g) were collected at monthly interval from Bonda, Panikhaiti, Panjabari, Khanapara, Jorabat and Gorchuk area of Guwahati, Assam according to the method described by Taylor [8], and the infective larvae (L_3_) was recovered according to the procedures outlined by Persson [9]. Nematode larvae (L_3_) were then concentrated into 20 ml water, and from each sample 1 ml subsamples were stained with Lugol’s iodine solution for identification using an Olympus BX51 light microscope at 100×, 200× and 400× magnifications according to the descriptions provided by Keith [10]. Larvae having rhabditiform esophagus were considered free-living nematode and not included in the counting. Counted larvae were then expressed as per kg dry matter of herbage (L_3_/kg DM) according to the method described by Sanyal and Gour [11].


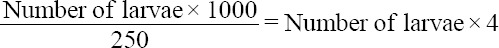


Again, the number of larvae per kg of DM was determined by finding out the DM content of infected pasture as per the standard method [12]. 100 g of pasture sample was weighed in a Petri dish and kept overnight in a hot air oven at 110°C. The DM content was calculated by the formula mentioned below.


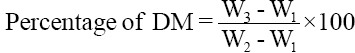


Where,

W_1_=Weight of petridish (g),

W_2_=Weight of petridish (g) + weight of pasture sample (before drying) in g

W_3_=Weight of petridish (g) + weight of pasture sample (after drying) in g

### Statistical analysis

Data were statistically analyzed using analysis of variance (ANOVA) for significance using SAS 9.3 facilitated under NAIP (Comp-I) from ICAR, New Delhi.

## Results and Discussion

**Figure-1:**
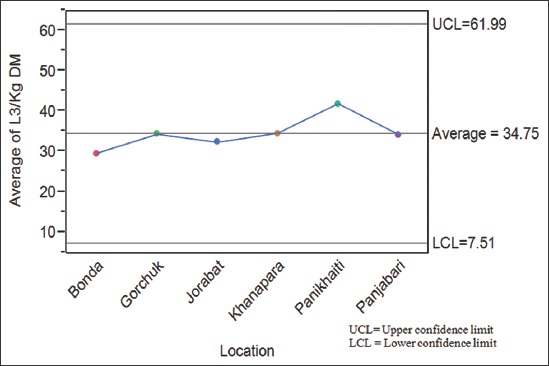
Average pasture larval burden (L_3_/kg dry matter) in six localities of Guwahati, Assam.

**Figure-2:**
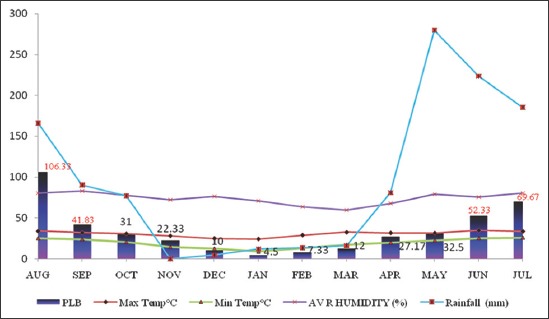
Correlation of meteorological parameters with pasture larval burden.

**Figure-3:**
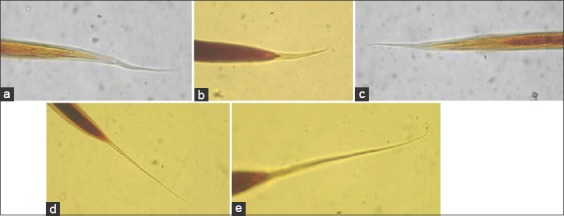
Posterior end of different nematode larvae (400×). (a) *Haemonchus* *contortus*, (b) *Trichostrongylus* sp., (c) *Mecistocirrus* spp., (d) *Cooperia* spp., (e) *Oesophagostomum* spp.

Pastures collected from six localities of Guwahati at monthly interval revealed presence of larvae in pastures throughout the year which varied from 4.5 L_3_/kg DM in January to a maximum of 106.33 L_3_/kg DM in August (Table-1). The average pasture larval burden (PLB) in Guwahati was 34.75±3.48 L_3_/kg DM (Figure-1). Season-wise PLB revealed the presence of 23.89±3.01, 67.54±5.41, 26.67±1.92 and 7.28±0.89 L_3_/kg DM during pre-monsoon, monsoon, post-monsoon and winter seasons, respectively (Table-2). Statistically monsoon season has significant (p<0.05) effect on PLB. However, ANOVA of different locations with respect to season revealed that there was no significant difference but season-wise it was highly significant (p<0.01) (Table-3). Correlation of environmental variables (temperature, relative humidity and rainfall) with PLB revealed that it was maximum during monsoon season (Figure-2). Pearson Correlation of environmental variables with PLB revealed correlation was statistically significant with rainfall at the 0.05 level (Table-4). The nematode larvae of *Haemonchus contortus*, *Trichostrongylus* spp., *Oesophagostomum* spp., *Cooperia* spp., and *Mecistocirrus* spp. were recovered from pastures (Figure-3).

**Table-1:** Month-wise PLB (L_3_/kg DM) in six localities of Guwahati, Assam.

Localities	August	September	October	November	December	January	February	March	April	May	June	July
Khanapara	116	46	27	15	7	5	4	13	20	34	59	72
Jorabat	112	40	30	20	4	5	6	17	22	27	42	66
Panjabari	100	38	36	28	15	3	10	10	30	35	50	59
Bonda	90	32	25	30	8	4	8	8	18	20	47	68
Panikhaiti	124	54	32	20	12	6	4	10	41	58	62	83
Gorchuk	96	41	36	21	14	4	12	14	32	21	54	70
Mean	106.33	41.83	31	22.33	10	4.5	7.33	12	27.17	32.5	52.33	69.67

DM=Dry matter, PLB=Pasture larval burden

**Table-2:** Season-wise PLB (L_3_/kg DM) in six localities of Guwahati, Assam.

Localities	(Mean±SE)				

	Pre-monsoon	Monsoon	Post-monsoon	Winter	Total
Khanapara	22.33±6.17	73.25±15.21	21±6.00	5.33±0.88	34.83±9.71
Jorabat	22±2.89	65±16.74	25±5.00	5±0.58	32.58±8.88
Panjabari	25±7.64	61.75±13.46	32±4.00	9.33±3.48	34.50±7.68
Bonda	15.33±3.71	59.25±12.63	27.5±2.50	6.67±1.33	29.83±7.65
Panikhaiti	36.33±14.05	80.75±15.66	26±6.00	7.33±2.40	42.17±10.46
Gorchuk	22.33±5.24	65.25±11.84	28.5±7.50	10±3.06	34.58±7.84
Mean±SE	23.89±3.01^ab^	67.54±5.41^c^	26.67±1.92^b^	7.28±0.89^a^	34.75±3.48

Letters with the same superscript are statistically at par P<0.05. DM=Dry matter, PLB=Pasture larval burden

**Table-3:** ANOVA (Location×Season) of PLB (L_3_/kg DM) in Guwahati, Assam.

Source	Sum of squares	df	Mean square	F	p value
Season	42299.49	3	14099.83	38.80	<0.01**
Location	701.2328	5	140.25	0.39	0.86^NS^
Season×Location	1188.264	15	79.22	0.22	0.99
Error	17444.08	48	363.42		
Total	61939.5	71			

** Significant at P<0.01. NS=Non-significant, DM=Dry matter, PLB=Pasture larval burden

**Table-4:** Pearson correlations (n=12) between environmental variables and PLB.

Environmental variables	PLB
Temperature °C (Max)	0.168^NS^
Temperature °C (Min)	0.268^NS^
Relative humidity (Av.)	0.329^NS^
Rainfall (mm)	0.473*

* Correlation is significant at the 0.05 level (2-tailed). NS=Non-significant, DM=Dry matter, PLB=Pasture larval burden

In the present findings, the pasture larvae burden in the grazing land of cattle were observed throughout the year and highest during monsoon season which was in conformity with the findings of Singh *et al*. [13], Bulbul *et al*. [14] and Singh *et al*. [15] from Madhya Pradesh, Assam and Rajasthan, respectively. Similarly, Kumar *et al*. [16] observed that the infective nematodes larvae in the grazing area were found at the onset of monsoon and continue to exist in the herbage but during the winter season the larval count decreases. Month-wise variations in the prevalence of infection in pasture were also in agreement with the findings of Al-Shaibani *et al*. [17]. In the present investigation, it was also observed that larval burden started declining from October, i.e., in the beginning of the dry season which was in accordance with the findings of Ogunsusi and Eysker [18] who suggested that in cattle and small ruminants of northern Nigerian origin, *Haemonchus* spp. survive in the host as arrested early fourth stage larvae during the long dry season with termination of the arrest at the beginning of the rainy season [19]. However, according to Durie [20] rainfall and temperature were probably more favorable for the development and survival of the pre-parasitic stages leading to increased availability of infective larvae on the pasture. Moreover, moisture also serves as a medium for the travel of larvae towards the tip of the forage so that they can reach to the grazing animals through the forages animal eat [21] viewed that the risk of infection is also lowered by not allowing animals onto pasture until the dew has lifted or the grass has dried after the rain. However, Van Dijk *et al*. [22] reported that the longevity of L_3_ larvae is not only related to temperature and humidity but also on exposure to ultraviolet light. The hot and humid climatic conditions of Assam are very congenial for propagation and perpetuation of parasites [23] so pasture management for animals is very crucial to keep the parasitic load at low level. Moreover, according to Stromberg and Averbeck [24], to design strategic parasite control program knowledge of the dynamics of egg shedding from the host and the pasture larval populations is required. It is important to know if larvae are available when animals are turned out onto pasture. Several steps can be practiced like stall feeding instead of open grazing to avoid contamination, particularly during rainy season. Rotational grazing and alternate species grazing are also advisable for control. Cutting the grass in the evening would also likely to reduce the chance of larval contamination because strongyle group of nematode larvae is positively phototropic.

## Conclusion

This study revealed that there is the presence of nematode larvae (L_3_) in the pastures of the grazing land of cattle throughout the year in Guwahati, Assam. Monsoon season has significant effect on pasture larval burden PLB and it is, therefore, necessary to properly manage the pasture and adopt suitable grazing practices to minimize the parasitic load.

## Authors’ Contributions

MD: Collected, processed and examined samples, prepared manuscript. DKD and SI: Interpretation of data, PCS: Data analysis, KB: Examined samples. All authors read and approved the final manuscript.
